# Quantitative investigation of a 3D bubble trapper in a high shear stress microfluidic chip using computational fluid dynamics and L*A*B* color space

**DOI:** 10.1007/s10544-024-00727-w

**Published:** 2025-01-13

**Authors:** Warisara Boonsiri, Hein Htet Aung, Jirasin Aswakool, Siraphob Santironnarong, Phattarin Pothipan, Rungrueang Phatthanakun, Wares Chancharoen, Aekkacha Moonwiriyakit

**Affiliations:** 1 Laboratory of Artificial Intelligence and Innovation in Medicine (AIIM), Princess Srisavangavadhana College of Medicine, Chulabhorn Royal Academy, 906 Kampangpetch 6 Rd., Talat Bang Khen, Lak Si, Bangkok, 10210 Thailand; 2 Defence Technology Institute, Office of the Permanent Secretary of Defence (Chaengwattana) 7th Floor, 47/433 Moo 3, Ban Mai, Pak Kret, Nonthaburi, 11120 Thailand; 3 Chakri Naruebodindra Medical Institute, Faculty of Medicine Ramathibodi Hospital, Mahidol University, 111 Suwannabhumi Canal Rd, Bang Pla, Bang Phli District, Samut Prakan, 10540 Thailand; 4 Synchrotron Light Research Institute, 111 University Avenue, Muang District, Nakhon Ratchasima, 30000 Thailand

**Keywords:** Microfluidic chip, COMSOL multiphysics, High shear stress, Bubble trapper, L*A*B* color space

## Abstract

Microfluidic chips often face challenges related to the formation and accumulation of air bubbles, which can hinder their performance. This study investigated a bubble trapping mechanism integrated into microfluidic chip to address this issue. Microfluidic chip design includes a high shear stress section of fluid flow that can generate up to 2.7 Pa and two strategically placed bubble traps. Commercially available magnets are used for fabrication, effectively reducing production costs. The trapping efficiency is assessed through video recordings with a phone camera and analysis of captured air volumes by injecting dye at flow rates of 50, 100, and 150 µL/min. This assessment uses L*A*B* color space with analysis of the perceptual color difference ∆E and computational fluid dynamics (CFD) simulations. The results demonstrate successful application of the bubble trap mechanism for lab-on-chip bubble detection, effectively preventing bubbles from entering microchannels and mitigating potential damage. Furthermore, the correlation between the L*A*B* color space and volume fraction from CFD simulations allows accurate assessment of trap performance. Therefore, this observation leads to the hypothesis that ∆E could be used to estimate the air volume inside the bubble trap. Future research will validate the bubble trap performance in cell cultures and develop efficient methods for long-term air bubble removal.

**Graphical abstract:**

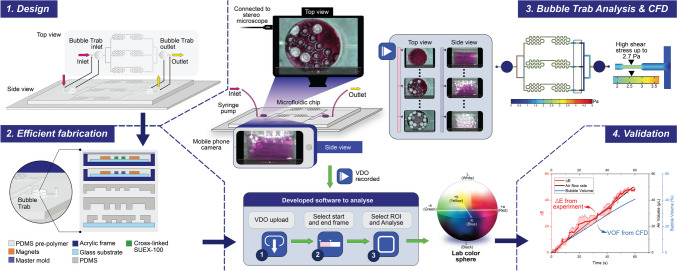

**Supplementary Information:**

The online version contains supplementary material available at 10.1007/s10544-024-00727-w.

## Introduction

Microfluidic technology is the design of fluidics at the millimeter scale where small amounts of fluids can be exactly controlled and integrated. These systems enable parallel, combinatorial, and sequential operations on a compact and flexible platform (Barata et al. 2017). Recent studies have used COMSOL Multiphysics simulations to determine fluid flow velocity and heat transfer profiles in 3D microfluidic devices (Kumar et al. 2013; Pisapia et al. 2022; Zhao et al. 2024a, b). The application of microfluidic technology to study the development of cells in a controlled setting has gained significant attention from the research community as the organ-on-a-chip platform. Among types of cells, osteoblasts, which are the important bone forming cells that are continuously exposed to and affected by mechanical stresses (Bartl 2023; Pisapia et al. 2022). Osteoblasts physiology and bone formation are determined high shear stress due to bone being a load-bearing organ, is constantly exposed to various mechanical stresses, such as blood flow, as well as physical tensions encountered during movement activities(Lochovsky et al. 2012). An important parameter is shear stress at the interface of flowing liquids and stationary solid phases, which has a influence on various microfluidic system especially in bone-on-a-chip application (Kim et al. 2018; Roy 2020; Xia et al. 2020)., Applied robust fluid flow and shear force prone the formation of tiny air bubbles within microchannels, often introduced during setup or due to dissolved gases (Park et al. 2021; Xiao et al. 2021). Understanding the influences of shear stress on osteoblast cells in microfluidic environments is crucial for optimizing chip design for bone cell development research. Apart from cellular applications, lab-on-a-chip technology has advanced in physics-based research. For example, precision fluid mixing and flow control must avoid bubble formation. Even in low flow system such as analytical chips still concern air bubble interference (Huang et al. 2020).

Kang et al. (2010) have identified one of the challenges in microfluidic chip design as being the formation and accumulation of bubbles, which can significantly affect system stability, cell viability, and experimental parameters (Kang et al. 2010; Sung and Shuler 2009). Bubble formation within the microfluidic channels might seriously threaten experimental accuracy and reliability, interrupt the flow field structure, increase fluid shear stress on cells, and cause cell death (Li et al. 2021b). Therefore, eliminating bubble formation is essential to ensure that microfluidic systems operate efficaciously. Several bubble trap designs have been developed to handle this problem, and they are generally classified into two approaches: passive traps and active traps (Gao et al. 2020). Passive bubble traps adopt the principles of natural fluid dynamics to trap and remove of bubbles while avoiding unnecessary energy consumption, an example being a 3D-printed cylindrical design tuned to rapid circulation (Markoski et al. 2021). These passive designs are often preferred for their simplicity and efficiency compared to active traps, which typically involve external mechanisms to actively remove bubbles (Narayanamurthy et al. 2020). Gao et al. (2020) found that the effectiveness and reliability of passive bubble traps significantly mitigate bubble-related issues within microfluidic systems. Moreover, comprehensive numerical analysis by Yang et al. (2021), including computational fluid dynamics (CFD), has provided a deeper understanding of the performance and efficiency of passive bubble traps. This analytical approach allows quantitative assessment of fluid flow dynamics, bubble capture efficiency, and system performance, providing valuable data to support the design and development of microfluidic systems. In summary, the development and use of passive bubble traps in microfluidic applications represent a strategic and practically effective approach. Advanced numerical analysis techniques such as CFD simulations can refine and optimize these passive solutions to address the critical challenge of bubble formation and accumulation within microfluidic environments(Gao et al. 2020; Yang et al. 2021).

Assessing image quality by reducing obscured air bubble fog begins with analysis in red, green, and blue (RGB) color space, which serves as a foundational framework for color examination. This color space defines the colors on the basis of the intensities of its three primary channels, red, green, and blue (Li et al. 2021b; Skelley and Voldman 2008). This method initially extracts color-based information and its distribution via RGB quantization within a given region of interest (ROI) and extraction of the corresponding color components of each pixel, thereby first characterizing the information and detecting variations in images containing trapped air bubbles (Lei et al. 2020; Sadashivan et al. 2021). However, RGB sensitivity to lighting variations can sometimes result in inaccuracies in color attributes under diverse environmental conditions. A method for improving image analysis to extract color information is moving the process from RGB color space to a color space based on hue, saturation, and value (HSV) (Kim and You 2024; Moreira et al. 2022; Sasidharan 2010). Unlike RGB, HSV separates color attributes into distinct dimensions: hue (H) represents the perceived color family, saturation (S) indicates color intensity or vividness, and value (V) describes brightness or lightness (Li et al. 2021a; Rani 2024). This property not only makes the transition well-suited for color analysis but also distinguishes air bubbles from the background fog or noise (Li et al. 2021a; Wang et al. 2023). In addition to RGB and HSV, the L*A*B* (CIELAB) color space is very useful for understanding colors accurately accordance with human vision as well as perception (Kaur and Kranthi 2012). The L*A*B* model defines colors on the basis of three perceptual dimensions: lightness (L*), which represents the perceptual brightness of the color green-red (A*), describing the color on a green to red axis and blue-yellow (B*), describing the color on a blue-yellow axis (Miao and Wang 2023; Patel and Patel 2019). Combining RGB, HSV, and L*A*B* color spaces provides a detailed method for color analysis during image provenance, especially air bubble detection and fog reduction.

The L*A*B* color space is a perceptually consistent color space. This is an efficient way to measure the perceived color difference between two images. We can compute the color difference between two images by computing the ΔE represents the difference between two colors in a three-dimensional color space. It is determined as the Euclidean distance between two points, where each point corresponds to a color in this 3D space. This concept is widely used in color science to quantify the perceived differences between colors. (Bautista et al. 2014; Yahyazadeh Shourabi et al. 2021). RGB is an important method because cell phone camera sensor is RGB so all images are natively RGB. That’s why we need to convert to other colour spaces and it creates HSV and L*A*B* color spaces by converting RGB color space to HSV color space using OpenCV on Python (Jung et al. 2022) and converting RGB to L*A*B* color space using the RGB to L*A*B* conversion equation (Baier et al. 2012).

The objective of this research is quantitatively evaluating the performance of this bubble trap using both CFD and L*A*B* color space. CFD allows detailed numerical simulations, providing insights into fluid behavior, flow patterns, and bubble dynamics within the microfluidic system. This numerical approach complements experimental findings by offering predictive capabilities and a deeper understanding of fluid dynamics. Furthermore, L*A*B* color values can be used to define variations in bubble accumulation and bubble reduction objectively, providing numerical data on color attributes that correlate with bubble presence and system stability. Combining these analytical methods, CFD for numerical fluid dynamics and L*A*B* color space for quantitative color analysis, enables a comprehensive evaluation of the air performance of the proposed bubble trap. This integrated approach aims to provide rigorous support for developing efficient and stable microfluidic systems with minimal bubble generation.

## Method

### Design of a microfluidic chip and bubble trap investigation using CFD

**Fig. 1:**
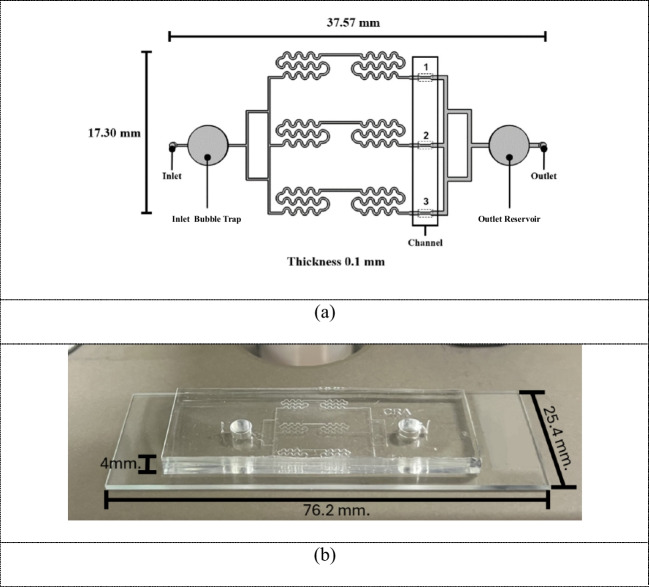
(**a**) Structure of a microfluidic chip designed in SolidWorks, where positions 1, 2, and 3 are the locations of the highest shear stress. The shear stresses at these three positions are nearly identical. (**b**) Polydimethylsiloxane (PDMS) microfluidic chip for applying high shear stress with two bubble trappers

The microfluidic chip was designed in three dimensions with the program SolidWorks Simulation 2020 (Dassault Systèmes SolidWorks Corp., France), and then the chip was simulated and analyzed with the program COMSOL Multiphysics Version 5.6 (COMSOL Inc., USA). The shear stress and air bubbles on the microfluidic chip were numerically analyzed under various flow rates to solve the Navier–Stokes equation regulating the movement of fluid and air bubbles. A fully developed flow was applied to the inlet of the microfluidic chip with steady flow rates of 50, 100, and 150 µL/min. No slip condition was enforced on the microfluidic wall channel. While it was observed that homogenous fluid, such as water, exhibited a slip wall characteristic, there was no study that documents a slip characteristic of fluid with mixture of air bubbles. Furthermore, the focus of this study was concentrated on bubble trap performance which is dominate by the inviscid flow and inherit fluid stability at low Re, the no slip condition at wall is more appropriate as shown by a good agreement with experimental results A constant pressure of 101,325 Pa was applied at both the inlet and outlet, as shown in Fig. 1(a) and (b). In Fig. 1(a), the three regions within the microfluidic chip identified as having the highest shear stresses are designated as regions 1, 2, and 3 as shown step file in the supplementary information. To enhance the fluid dynamics within the microfluidic chip, we integrated a serpentine structure prior to the working zone. This design was specifically chosen to ensure laminar flow throughout the entire working zone, thereby minimizing the possibility of turbulent disturbances that could interfere with the fluid’s behavior and shear stress. Additionally, we have integrated the use of inlet bubble trap and outlet reservoir into our conventional microfluidic design. The inlet bubble trap captures any air bubble, that might enter the system, before it reaches the working zone. This prevents bubbles from interfering with the laminar flow within the chip. On the other hand, the outlet reservoir is designed to allow liquid to drain quickly from the working zone and designed to prevent back flow current and air bubbles to re-enter the system disrupting flow consistency (Bahmaee et al. 2020). Additionally the use of simple geometric shape, a cylinder, as the bubble trapping mechanism allows for more straight forward simulation in the CFD software.

The Reynolds number is a dimensionless measure that compares the viscous forces to the inertial forces in a fluid. It is usually used a criterion to define whether the fluid flow is turbulent or laminar. The Reynolds number for an internal flow with a rectangular cross section can be calculated according to (Bahmaee et al. 2020).

1 $$\:Re\:=\:\frac{2\rho\:Q}{\left(w+h\right)\mu\:}$$

where $\:\rho\:\:$ is the fluid density (0.001 g/mm^3^ for water), $\:Q\:$ is the flow rate (µL/min), $\:h\:$ and $\:w\:$ are the height and width (mm) of channel, and $\:\mu\:\:$is the dynamic viscosity of the fluid (8.90 × 10^−4^ Pa·s). In this study, the maximum Reynolds number is 0.01 at a flow rate of 150 µL/min, far below the critical Reynolds number of 2,300 for internal flow. Thus, the flow is expected to be laminar, and the laminar Navier–Stokes equation model was selected for the calculations. Numerical grid was generated using 82,405 tetrahedral elements. Maximum element growth rate was kept below 1.15 to ensure a smooth transition and minimize numerical error.

**Fig. 2:**
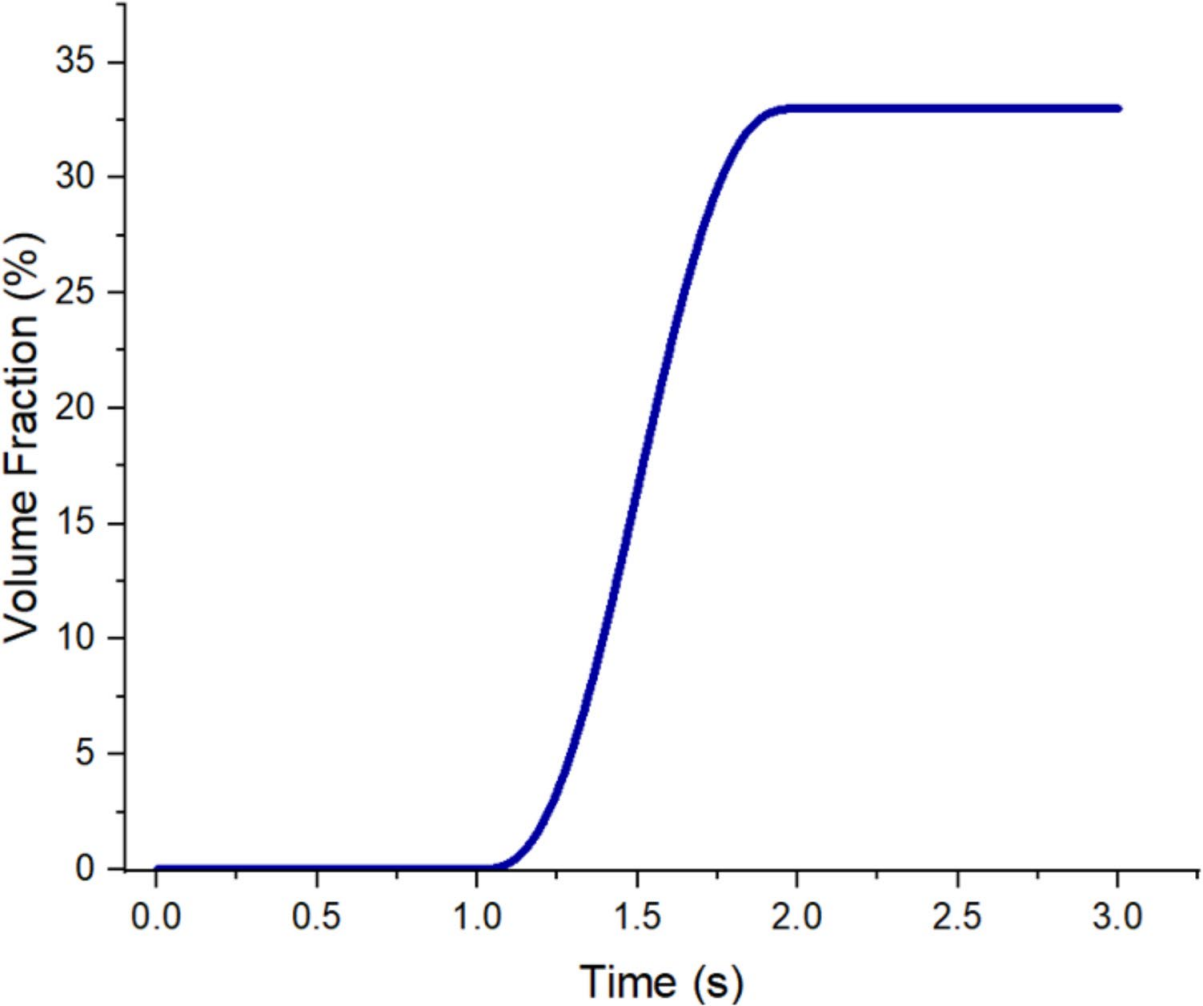
Ramp function of dispersed phase concentration during initial simulation

The transport and concentration of air bubbles (dispersed phase) in water (continuous phase) were calculated using a laminar mixture model assuming the densities of both phases are approximately constant. The density and the dynamic viscosity of air are 1.2 × 10^−6^ g/mm^3^ and 1.81 × 10^−5^ Pa·s respectively. This assumption was satisfied because the flow rate in the microfluidic chip was sufficiently low. Furthermore, the condition of conservation of the dispersed phase was imposed. Thus, the size of the dispersed phase may change but not vanish or merge. The relative velocity between the two phases was computed using the Haider–Levenspiel model (Haider and Levenspiel 1989), which is suitable for non-solid dispersed phase particles. The time-dependent Navier–Stokes equation with a laminar mixture model was solved using the finite element method (FEM). To aid the convergence and mimic the experiment, the simulation was initiated with no air bubbles to establish a steady flow field in the microfluidic chip. The concentration of the dispersed phase or Volume Fratction of air, define as VOF_air_ = (Volume of air / Volume of air & water), was introduced 1 s after the simulation started and slowly ramped up toward the final desired value within 3 seconds after the simulation was initiated. Figure 2 shows the ramp function of the dispersed phase concentration simulated using COMSOL Multiphysics, and the detailed data supporting this simulation are provided in the supplementary information.

### Fabrication

The layout of the microfluidic chip was first designed using Layout Editor (Juspertor GmbH, Germany) to provide a negative photomask for photolithography with a 100 μm thickness with a black ink pattern. A 100 negative photoresist sheet (SUEX-100, DJ MicroLaminates Inc, United States) was placed on the substrate and laminated with a hot roll laminator (304 Hot Roll Laminator, Fortex, United Kingdom) at a speed of 0.16 m/min at 70 °C. The photoresist was deposited on the substrate photomask and placed on the top and exposed to 395 nm UV light (Mask Aligner EVG610, EV Group, Austria) with a total dose of 2700 mJ/cm^2^. For pre- and post-baked UV exposure, a post-exposure bake (PEB) was performed in two steps, at 65 °C for 5 min and 95 °C for 15 min. The part of the photoresist exposed to UV light was not soluble in a developed chemical [2-(1-Methoxy) propyl acetate 99%] (Acros Organics, Germany) and remained permanently after the development process to obtain a master mold.

For the fabrication of the bubble-trapping master mold, a two-layer structure was utilized. The first layer, 100 μm thick, consists of microchannel structures placed on the substrate, while the second layer, 4 mm thick, is applied at the inlet and outlet areas. The bubble-trapping design is based on the principle of capturing bubbles that flow into the microchannel. Therefore, the bubble tank was designed with a diameter of 2 mm. Adjacent to the inlet and outlet, there are two circular patterns with a diameter of 2 mm, forming the base for bubble trapping, which supports the second layer. To conserve photoresist material for the second layer, two cylindrical neodymium magnets, each 2 mm in diameter and 4 mm in height, were placed on the bubble-trapping base areas during the preparation of the master mold for PDMS replication.

A key consideration here is that there is no gap left in the positive feature of the master mold where the magnets are placed.To prevent PDMS leakage during the pour and baking steps, we ensured that the magnets formed a tight seal with the base area. The strong magnetic force between the magnets and the metal plate beneath the mold ensured that the magnets were securely pressed against the photoresist surface, eliminating any potential gaps. This tight attachment prevented any pre-curing PDMS from leaking into the junction during the casting process. After curing, the magnets could be easily removed from the cured PDMS without disrupting the structure, and they can be reused multiple times for future mold preparations.

**Fig. 3:**
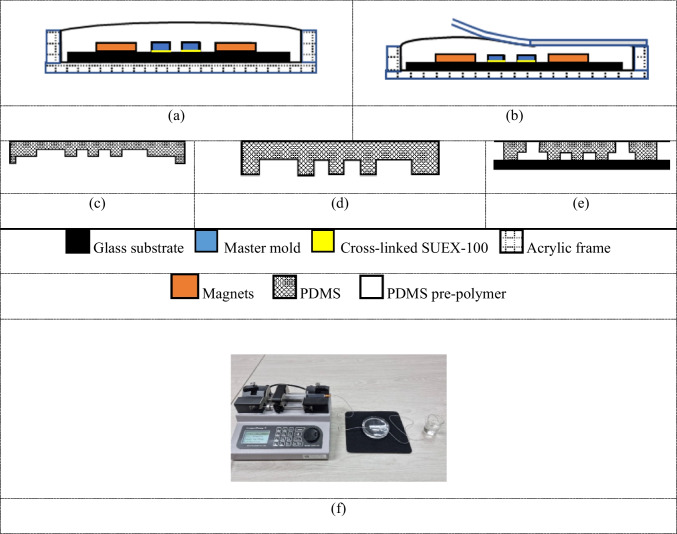
Fabrication process of a microfluidic chip using polydimethylsiloxane (PDMS) soft lithography. (**a**) PDMS is poured into the acrylic block at the position of the magnet bubble trapping zones after the entrance and before the exit in the microfluidic chip. (**b**) Pouring of PDMS into the acrylic block is completed. (**c**) The PDMS mold is peeled off. (**d**) PDMS mold is cut. (**e**) Plasma bonding is performed between glass slides with PDMS. (**f**) The laboratory setup for testing microfluidic chip leakage is completed

The master mold made from the photoresist was next fabricated via soft lithography. Polydimethylsiloxane (PDMS) (Dow Chemical, Germany) was mixed with a ratio of 10:1 and poured into the prepared acrylic block. Magnets were placed in each of the two acrylic sheets, as shown in Fig. 3(a). Then, magnetic bars were placed on both sides of the bubble trap, as shown in Fig. 3(b). The acrylic frame was heated in an oven (Lenton, Carbolite Gero, United Kingdom) at 70 °C for 60 min, and the PDMS was then slowly peeled off the master mold, as shown in Fig. 3(c) and (d). Finally the PDMS chip was punched using a PDMS Microfluidic Chip Hole Puncher (Elveflow, France) to form entrance channels connected to the inlet and outlet locations; and then it is attached to the glass surface using oxygen plasma bonding (Electronic diener, Baden-Württemberg, Germany) at a power of 150 W and a pressure of 150 mTorr for 60 s. After plasma treatment, the PDMS components were gently pressed together for 30 s, creating a microfluidic chip. Figure 3(e) and 1(b) show a complete PDMS microfluidic chip surrounded by a sub-glass state. Stainless steel 90° bent couplers (Darwin Microfluidics, France) were then placed inside the punched holes to allow connection to the syringe pump.

We performed leak testing of fabricated microfluidic chips using a 5 mL syringe connected to a syringe pump, operating at a flow rate of 100 µL/min. Gas-based leakage detection methods, using external visual observations and internal sensing techniques, have distinct advantages over traditional liquid-based testing approaches. Figure 3(f) shows our experimental setup, with a tube connecting the inlet and outlet of the microfluidic chip, with water as the test medium. The presence of air bubbles observed during testing serves as a reliable indicator of a leak-free microfluidic chip (Silverio et al. 2022).

**Fig. 4:**
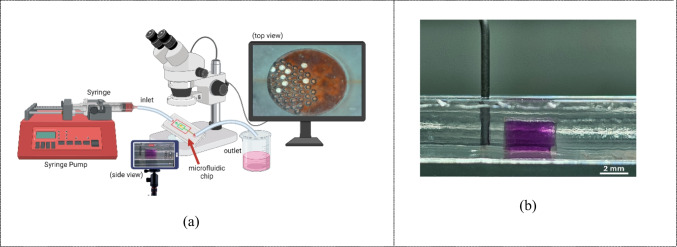
(**a**) Experimental setup for recording video of the side and top of the bubble trap. (**b**) Side view of the bubble trap in the microfluidic chip

After the leakage test was completed and the PDMS microfluidic chip was cleaned with 70% alcohol, the equipment was prepared for testing the bubble trap. A 5 mL syringe, containing a mixture prepared with 51.8 mg of potassium permanganate, 200 mL of deionized water, and a 500 mg surfactant solution, was connected to a syringe pump with an input flow rate set to 50 µL/min. Tubes were attached to the outlet and inlet of the microfluidic chip, which was then connected to a beaker and syringe pump respectively. Bubbles within the microfluidic chip were observed using a stereo microscope (Leica DM1000-3000 LED, IDM Instruments, Australia) at the top of the bubble trap with a 10X magnification, as shown in Fig. 4(a), and the side of the bubble trap was observed using a smartphone camera, as shown in Fig. 4(b).

### Bubble trap investigation on a microfluidic chip using L*A*B* color space

**Fig. 5:**
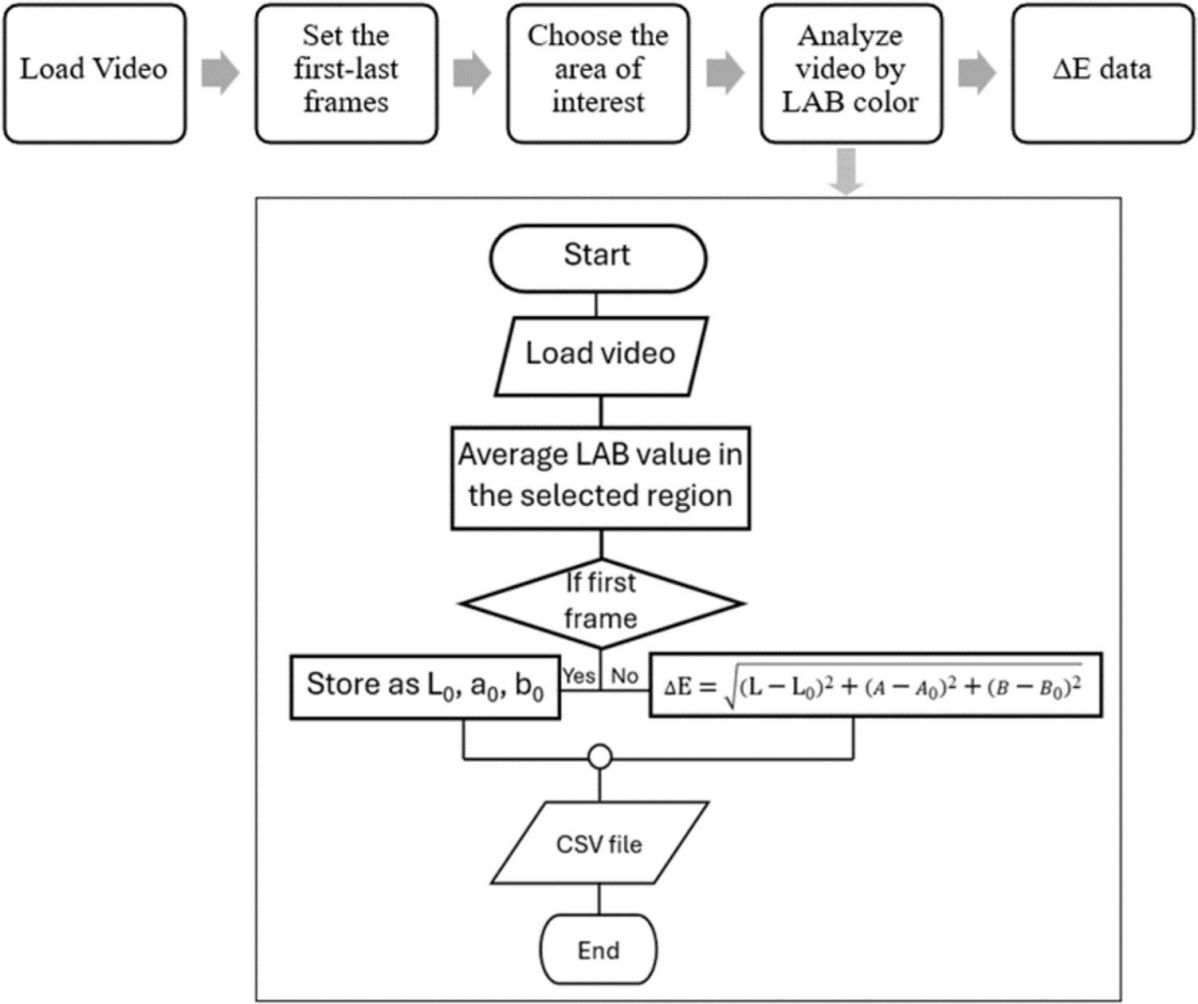
Data analysis flowchart. The process starts with uploading the video into the MATLAB program, and then determining the number of frames and selecting the area of interest to measure. The program stores the values L_0_, a_0_, and b_0_ and then calculates them using ∆𝐸, and the result comes out as a CSV file

The video analysis application developed in MATLAB serves as a comprehensive tool for evaluating colorimetric changes in video sequences. This application is designed to allow fine-grained analysis over the video contents using L*A*B* color space, known for its perceptual uniformity and effectiveness in representing human vision. The application allows users to load their video files and manually select ROIs within the first frame. It then performs an automated analysis over specified frame ranges, extracting and plotting L*A*B* color values for each frame within the region of interest (ROI). The perceptual color difference ∆E between frames is calculated. The L*A*B* color values and ∆E measurements are visualized in real time with an option to save the analyzed data to a CSV file, as shown in Fig. 5.

The method proposed to measure the fluid level inside the installed bubble trap in a microfluidic chip involves image processing techniques to calculate the light intensity passing through the fluid. The intensity of light passing through the fluid is related to the fluid level. Image processing is the method of measuring the light intensity using a smartphone camera (Samsung S23 ultra, Samsung Electronics Co., Ltd., South Korea) to record the level of the fluid image. This method applies to measuring the fluid inside the bubble trap, and this can be used to study the fluid behavior and air bubble relationship using L*A*B* color space.

The bubble trap image was recorded with a smartphone camera on the RGB color base showing visually observable changes in the level of fluid inside the bubble trap. Initially, the captured image in the RGB color codex was converted to the HSV color base for preliminary analysis, transforming the 3-dimensional vector color space to the HSV color space (Akiba and Tanaka M 2020; Bautista et al. 2014). However, in the preliminary analysis, the results of the individual channels in HSV analysis were inconclusive due to fluctuations in the graph and there is not meaningful way to combine the 3 dimensions of HSV. Consequently, the analysis shifted from RGB to L*A*B* color space using MATLAB program, as shown in the supplementary information.

The color conversion is fundamental process in color science and imaging, allowing the transformation of color representations from the device-dependent RGB space to the device-independent xyz space. Subsequently, the xyz values serve as the basis for further conversion to the L*A*B* color space, which prepares perceptually uniform color representations. This approach ensures accurate and consistent color analysis. The first step is converting from RGB to xyz with the Eq. 

2 $$\:\left[\begin{array}{c}x\\\:y\\\:z\end{array}\right]=\left[\begin{array}{ccc}0.4124&\:0.3576&\:0.1805\\\:0.2126&\:0.7152&\:0.0722\\\:0.0193&\:0.1192&\:0.9505\end{array}\right]\left[\begin{array}{c}R\\\:G\\\:B\end{array}\right]$$

Let 𝑅, 𝐺, and 𝐵 be the normalized RGB values scaled to the range [0,1]. The linear RGB color values (R, G, B) of each pixel are first mapped into the xyz color space. These equations normalize the RGB values on the basis of their sum to obtain the xyz tristimulus values (x, y, z) of the pixel. The matrix coefficients for converting normalized RGB to xyz are derived from the CIE 1931 standard observer, which is a mathematical model representing the average spectral sensitivity of human vision. These coefficients are based on empirical data obtained from experiments involving human observers and are standardized by the International Commission on Illumination (CIE) (Akiba and Tanaka 2020; Magnusson et al. 2020). The second step is to convert from xyz to L*A*B* using (Baier et al. 2012)

3 $$\:\begin{array}{c}{\:\:L}^{*}=116f\left(\frac{y}{{y}_{0}}\right)-16\:,\:\:\:\:\:\:\:\:\:\:\:\\\:{\:A}^{*}=500\left[f\left(\frac{x}{{x}_{0}}\right)-f\left(\frac{y}{{y}_{0}}\right)\right]\:,\\\:{\:B}^{*}=200\left[f\left(\frac{y}{{y}_{0}}\right)-f\left(\frac{z}{{z}_{o}}\right)\right],\end{array}$$

where,

$$\:\:\:\:\:\:\:\:\:\:\:\:\:\:\:\:\:\:\:\:\:\:\:\:\:\:\:\:\:\:\:\:\:\:\:\:\:\:\:\:\:\:f\left(x\right)=\left\{\begin{array}{c}{x}^{1/3},\:\:x>0.00886\\\:7.787x+\:\frac{16}{116},\:\:x\le\:0.00886\end{array}\right.$$

is derived from the CIE 1931 xyz color space and is designed aims to replicate the nonlinear response of human vision to light intensity. It is used to normalize the xyz values before calculating the L*A*B* values. The purpose of this normalization is to ensure that the resulting L*A*B* values correspond accurately to the colors perceived by human observers (Akiba and Tanaka 2020; Magnusson et al. 2020).

∆E is the perceptual color difference measured from the L*A*B* color space; it is used to determine the difference between two colors that visually defines an image. It is calculated from the L*A*B* color values of two points or two images that are compared. Calculating ∆E values helps us understand the degree of difference in color that humans can perceive with the naked eye. ∆E can be calculated using the formula for the Euclidean distance between the L*A*B* values of two points. The commonly used formula is CIEDE2000 (CIE 2000) (Gómez-Polo et al. 2020), which is a highly accurate method for estimating the difference between two colors or color differences between two images. The xyz tristimulus values (x, y, z) obtained from the first step are then used to calculate the L*A*B* color values, where x_0_, y_0_, and z_0_ are the tristimulus values of the reference. These equations transform the xyz tristimulus values into the perceptually uniform L*A*B* color space. The CIEDE2000 formula for calculating ∆E from the L*A*B* values is as follow (Bautista et al. 2014):

4 $$\:\:\:\:\:\:\:\:\:\:\:\:\:\:\:\:\:\:\:\:\:\:\:\:\:\:\:\:\:\:\:\:\:\:\:\:\:\:\:\Delta\:\text{E}=\sqrt{\left(\text{L}-{\text{L}}_0\right)^2+\left(A-A_0\right)^2+\left(B-B_0\right)^2}\:\:\:\:\:\:\:\:\:\:\:$$

L* is associated with brightness, A* with the red-green color, and B* with the yellow-blue color.

For the proposed analysis method, the average L*A*B* value and consequently the ∆E for each frame is calculated by converting the RGB value of each pixel in the area of interest to L*A*B * and computing the mean L*, mean A* and mean B* values, as shown in Fig. 5. The analysis starts with the selection of the video file; it is important to ensure the field of view remains the same for the entirety of the video, without any movement of camera, for the code we implemented. To ensure start and end of the analysis align with the start and end of fluid flowing into microfluidic chip, respective the frames in the video must be defined. Then an area of interest should be selected to include the entire or majority of the pixels of the bubble trap image in the video. The color value of each frame is averaged over this area, ensuring that local color changes (such as a small bubble appearing) does not affect the evaluation of the entire bubble trap. Using Eq. (4), the analysis of the video calculate the color change of each individual from to the first frame and outputs the color change value(∆E) array with time index, whereL_0_, A_0_, and B_0_ are the color values in the first frame and L, A and B are color values of the current frame.

## Results and discussion

### Qualitative analysis using CFD

#### Shear stress simulation

**Fig. 6:**
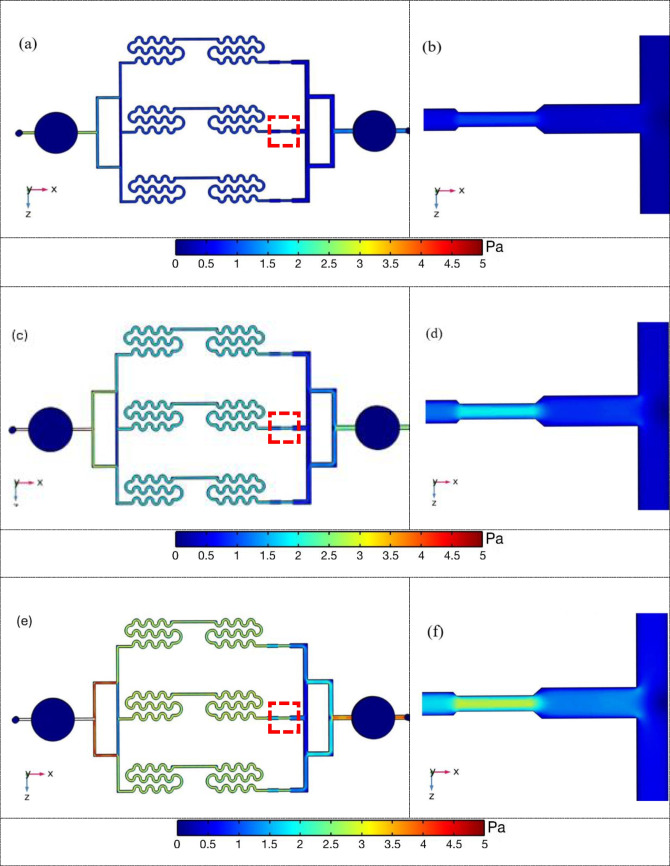
Shear stress throughout the microfluidic chip. (**a**) Stress distribution and (**b**) maximum shear stress area for a flow rate of 50 µL/min. (**c**) Stress distribution and (**d**) maximum shear stress area for a flow rate of 100 µL/min. (**e**) Stress distribution and (**f**) maximum shear stress area for a flow rate of 150 µL/min. The three high shear stress positions are those in Fig. 1(a)

The chip was simulated in COMSOL Multiphysics for inlet flow rates of 50, 100, and 150 µL/min. The simulation indicated a maximum streamline velocity of 45 × 10^−3^ m/s within the chip. Streamline motion within the microfluidic chip was also observed. Moreover, the pressure distribution throughout the microfluidic chip varied from 0 to 365.15 Pa. The pressure behavior within the chip was analyzed to understand the flow characteristics. Subsequently, the shear stress distribution throughout the microfluidic chip was examined. The shear stress was found to vary significantly at different flow rates. At flow rates of 50, 100, and 150 µL/min, the maximum shear stresses observed were 0.90 Pa, 1.85 Pa, and 2.70 Pa, respectively, as shown in Fig. 6(a)–(f). This provides crucial insights into fluid flow behavior within the microfluidic environment, highlighting the relationship between flow rate and shear stress, which is critical for optimizing chip performance and ensuring the integrity of the flow conditions for various applications.

#### Volume fraction (VOF) simulation

**Fig. 7:**
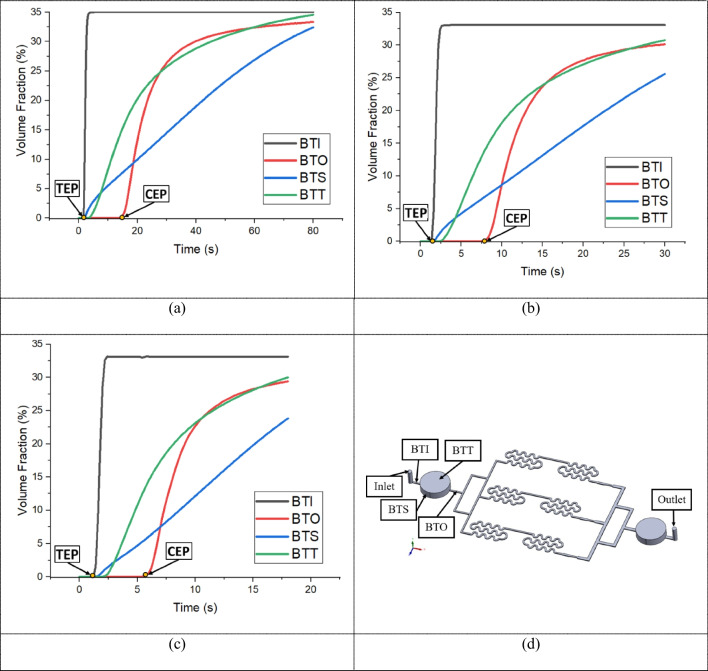
Average volume fraction (VOF) of bubbles at the inlet, side, top, and outlet surfaces of the bubble trap for flow rates of (**a**) 50 µL/min, (**b**) 100 µL/min, and (**c**) 150 µL/min. (**d**) Bubble trap inlet (BTI), bubble trap side surface (BTS), bubble trap top surface (BTT), and bubble trap outlet (BTO) of the bubble trap used to measure the average VOF of the bubbles. Figure 7(**a**)-(**c**) also indicate the trap entry point (TEP) and channel entry point (CEP) times, as shown in Fig. 8(**a**)–(**f**)

**Fig. 8:**
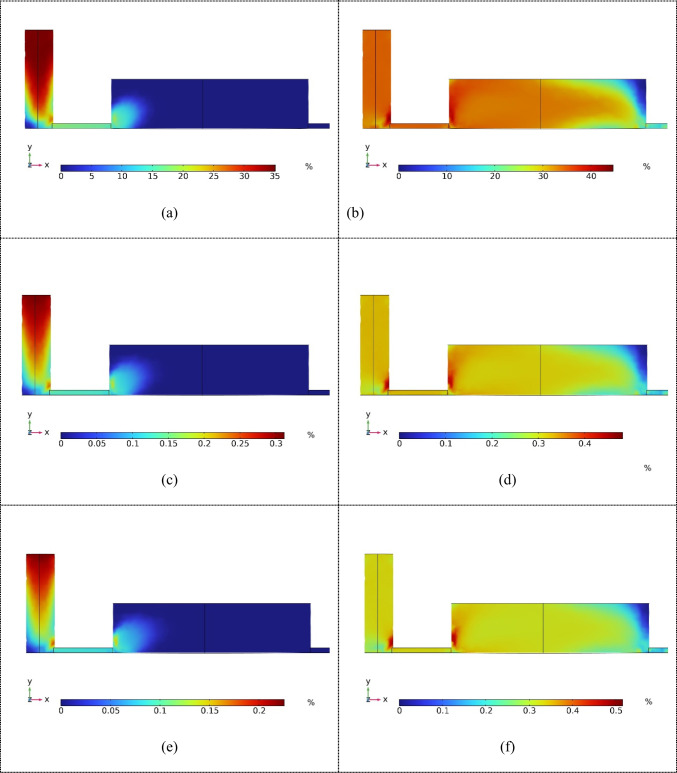
Centerline planes for contours. (**a**) VOF contour of TEP at a flow rate of 50 µL/min at 2.2 s. (**b**) VOF contour of CEP at a flow rate of 50 µL/min at 15 s. (**c**) VOF contour of TEP at a flow rate of 100 µL/min at 1.8 s. (**d**) VOF contour of CEP at a flow rate of 100 µL/min at 8.2 s. (**e**) VOF contour of TEP at a flow rate of 150 µL/min at 1.6 s. (**f**) VOF contour of CEP at a flow rate of 150 µL/min at 6.2 s

The performance of the bubble trap introduced in the microfluidic chip was assessed through detailed VOF analysis for air bubbles at various critical locations. In COMSOL, the VOF method tracks the percentage of air (vol: vol) present in each computational cell in. This allows for detailed analysis of air distribution within the bubble trap. By monitoring the volume fraction of air as time progress, the effectiveness of the bubble trap can be assessed as air move through the chip different part of the chip at different flow rates. Figure 7(a)–(c) shows the VOFs at the bubble trap inlet (BTI), bubble trap side (BTS), bubble trap top (BTT), and bubble trap outlet (BTO) surfaces, as labeled in Fig. 7(d). The plots highlights the trap entry point (TEP), which is the time when the bubbles begin to enter the bubble trap, and the channel entry point (CEP), which is the time when bubbles begin to enter the channel at flow rates of 50, 100, and 150 µL/min. Figure 7(a-c) shows that compared at 150 µL/min the bubble trap saturates in the shortest time compared to 50 and 100 µL/min, additionally the time points of TEP and CEP of different flow rate indicate that at slower the flow rate is the longer it takes for the bubbles to appear at the outlet of the bubble trap outlet (BTO). This multi-point analysis allows a detailed understanding of how bubbles behave and are captured within the trap under different flow conditions. This analysis is essential for understanding the aspects of bubble formation, capture, and elimination. By plotting these times, we can observe the fluctuations in bubble concentration and how quickly the trap stabilizes the system with the four VOF curves converging. Figure 8(a)–(f) show the air bubble TEP and CEP contours from the bubble trap VOFs for the three flow rates, capturing the dynamic behavior of bubbles over time.

Figure 8(a)–(f) illustrate the evolution of air bubble concentration in the bubble trap as a function of time, which is reflected by the air bubble VOF. Each subfigure represents a different time interval in which the flow profile of air bubble aggregation develops throughout the experiment. At higher flow rates, the bubble trap saturates faster. This is evidenced by the increase in air bubble VOF at the outlet of the bubble trap, which marks the point when the trap can no longer contain additional bubbles and they start to exit. Figure 8(a)–(f) reveal a locally higher air bubble VOF especially around the trap sides and corner with respect to the decreased flow at elevations overlying those regions. This can be attributed to the accelerated flow in these regions, which leads to a reduction in local pressure. The decreased pressure promotes the air bubble formation, thus explaining the higher VOF observed in these areas.

**Fig. 9:**
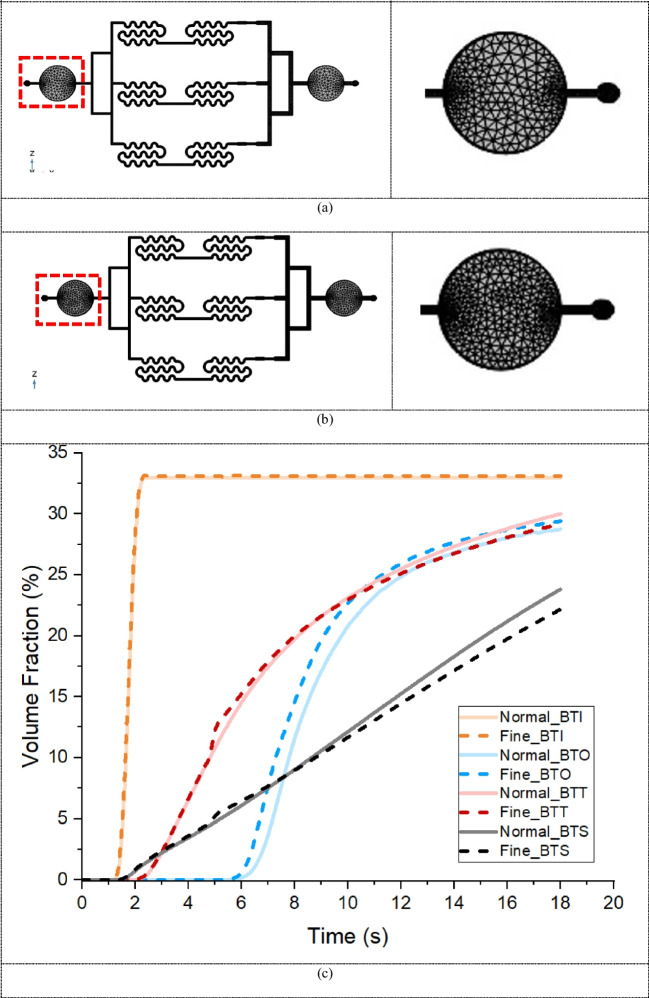
Overall views of normal and fine computational meshes. (a) The normal mesh has 45,558 elements and a size of approximately 5.7 × 10^−5^ to 4 × 10^−4^ m. b) The fine mesh has 82,405 elements and a size of approximately 1.15 × 10^−5^ to 3.8 × 10^−4^ m. (c) Air bubble concentration for both fine and coarse meshes at the inlet (orange line), outlet (blue line), top (red line), and side (black line) surfaces of the bubble trap. The line graph represents the coarse mesh, while the dashed line graph corresponds to the fine mesh

The resulting air bubble VOFs at BTI, BTS, BTT and BTO of the bubble trap are presented in Fig. 9(a)–(c). The numerical mesh used in this study is an unstructured mesh that can be generated on complex geometry. A mesh sensitivity study was performed by conducting a mixture multiphase flow at a flow rate of 150 µL/min on both normal and fine meshes. The resulting air bubble VOFs for both coarse and fine meshes agree well. The air bubble concentrations on the bubble trap surface as well as at BTI, BTS, BTT and BTO show similar distributions for both mesh types. This indicates that the mesh resolution we used is adequate for capturing the essential flow characteristics. However, a fine mesh was selected to further reduce numerical errors and diffusion effects. This ensures that the solution flow field is minimally altered, thus maintaining computational efficiency without compromising accuracy. The similarity in the curves indicates that the mesh resolution is adequate to accurately capture the necessary flow characteristics. Additionally, the solution flow field exhibits minimal variation, confirming the reliability of the chosen mesh resolution.

### Quantitative analysis using L*A*B* color space

**Fig. 10:**
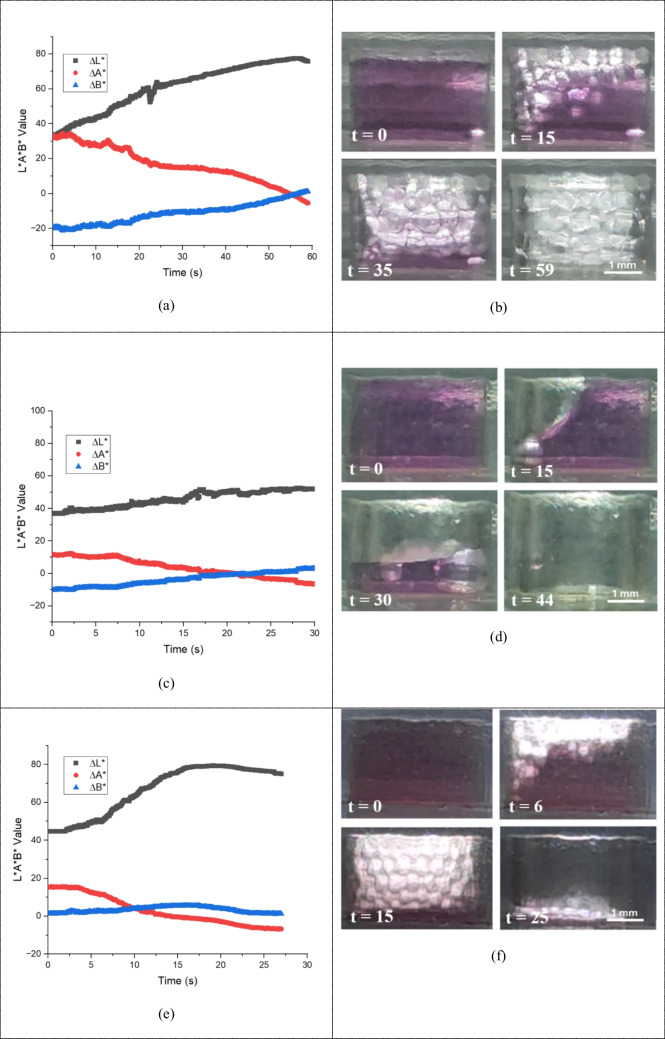
Graphs of perceptual color difference ∆E from color analysis in the bubble trap in L*A*B* color space, where the black line is lightness L*, the red line is the green-red axis A*, and the blue line is the blue-yellow axis B*. (**a**), (**c**), and (**e**) show graphs of L*, A*, and B* values at flow rates of 50, 100, and 150 µL/min respectively, and (**b**), (**d**), and (**f**) show images of the bubble trap during L*A*B* analysis in seconds timeframe

The initial experimental condition, marked by the maximum light intensity, represents the original volume of colored water, reflecting the maximum color saturation. At this stage, the lightness (L*) values are generally very low owing to the high density of the colorant, which results in a predominantly dark appearance (t = 0 s). When air bubbles are introduced into the colored water, they disperse throughout the liquid, creating white regions, thereby increasing the overall lightness (L*) of the mixture. Consequently, the color appears lighter and less saturated. Concurrently, the color coordinates A* and B*, which represent the red-green and yellow-blue axes, also undergo minor variations. The A* values decrease as the pink hue diminishes with the diluting effect of the bubbles. This reduction in the red component leads to a less pronounced pink coloration. However, the B* values remain relatively stable with only slight fluctuations. These minor changes in the B* coordinate can be attributed to the influence of the background color interacting with the dispersed bubbles. Moreover, the rate of color change is influenced by the flow rate at which the air bubbles are introduced. At higher flow rates, bubble dispersion occurs more rapidly, leading to quicker increases in L*, A*, and B* and therefore faster changes in the color coordinates. This dynamic fluid flow and bubble distribution within the system play a crucial role in the observed variations in color properties, as shown in Fig. 10(a)–(f).

### Validation of CFD and L*A*B* color space results

**Fig. 11:**
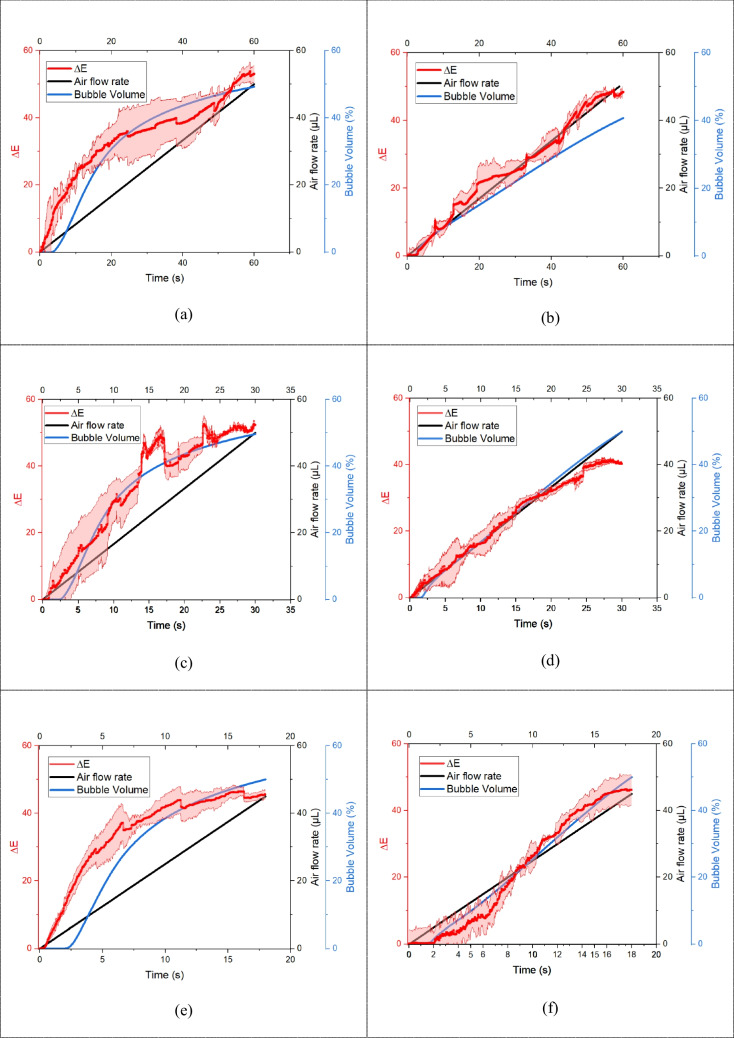
Comparison of results for ∆E (red line), air flow rate (black line), and bubble volume (blue line). (**a**), (**c**), and (**e**) illustrate the top surface results for 50, 100, and 150 µL/min, respectively, while (**b**), (**d**), and (**f**) depict the side surface results for the same flow rates. The red shading indicates the standard deviation of ∆E

The results were collected by observing the trends of ∆E, air flow rate, and volume fraction at the side and top of the bubble trap. The VOF method from CFD was employed to model the dynamics of air bubbles. within the microfluidic system and ∆E obtained from experiment. Both experimental and simulated results indicated a consistent increase in ∆E (red line) and bubble volume (blue line) in Fig. 11(a)–(f) along the same direction, confirming the effectiveness of the bubble trap in capturing air bubbles. We discovered that the color brightness and saturation, expressed as ∆E, were representative of the air volume inside the bubble trap. At maximum brightness, the color approaches white, signifying a high bubble volume. The data revealed that ΔE at the side of the bubble trap was consistently higher than the measured air volume under all conditions.

Therefore, this observation led to the hypothesis that ∆E could be used to estimate the air volume inside the bubble trap. CFD simulations were conducted to validate the experimental findings. The simulations included all the experimental conditions, providing a comparative dataset. By plotting ∆E at the top and side of the bubble trap with VOF, we observed a strong correlation between the two datasets. This correlation confirmed that ∆E is a reliable estimator of the actual air volume inside the bubble trap. The primary advantage of using ∆E as a metric lies in its simplicity and ease of measurement, which negate the need for more complex or invasive techniques. This method significantly enhances the efficiency and accuracy of monitoring and controlling microfluidic systems.

## Conclusions

In this research, a bubble trap was used in a microfluidic chip to lessen the disruptive influence of air bubbles. A combination of CFD models and experimental observations validated the bubble trap performance. Key metrics, including ∆E, air volume, and VOF, were analyzed across air bubble flow rates of 50, 100, and 150 µL/min to validate the bubble trap efficiency. L*A*B* color space analysis was shown to be a dependable, economical technique for determining air volume and eliminating bubbles in microfluidic devices. Strong correlation between L*A*B* color space data and CFD-derived VOF metrics validated the bubble trap efficiency. Meanwhile, the study highlighted three high shear stress locations, with a maximum stress of 2.7 Pa. However, this microfluidic chip has limitations regarding the storage capacity for air bubbles, which can hold a maximum of 50 µl. Additionally, when recording video for color analysis, it is essential to arrange consistent lighting across all conditions to ensure standardization of the experiments. Future experiments will focus on further validating the bubble trap performance in real-world biological applications. Efforts will also be directed towards developing efficient methods for removing accumulated air bubbles from the bubble trap to ensure sustained optimal performance over extended periods.

## Electronic supplementary material

Below is the link to the electronic supplementary material.

Supplementary Material 1

## Data Availability

All data generated or analyzed during this study are included in the supplementary materials.

## Declarations

### Author declaration

During the preparation of this article, the authors used generative AI to refine sentence structures, grammar, and overall language quality. After using this tool, the authors reviewed and edited the content as needed and take full responsibility for the content of the publication.
